# Tranexamic acid dosing in pediatric trauma: Dose simulation based on population pharmacokinetic modeling in adult trauma patients

**DOI:** 10.1111/trf.70047

**Published:** 2025-12-23

**Authors:** Gideon Stitt, Kevin Downes, Athena Zuppa, Christine Leeper, Kevin Watt, Philip Spinella

**Affiliations:** ^1^ Division of Clinical Pharmacology, Department of Pediatrics University of Utah Salt Lake City Utah USA; ^2^ Center for Clinical Pharmacology Children's Hospital of Philadelphia Philadelphia Pennsylvania USA; ^3^ Division of Infectious Diseases Children's Hospital of Philadelphia Philadelphia Pennsylvania USA; ^4^ Department of Pediatrics, Perelman School of Medicine University of Pennsylvania Philadelphia Pennsylvania USA; ^5^ Department of Surgery and Critical Care Medicine University of Pittsburgh Pittsburgh Pennsylvania USA; ^6^ Division of Pediatric Critical Care, Department of Pediatrics University of Utah Salt Lake City Utah USA

**Keywords:** emergency medicine, hemorrhage, hemostasis, pediatrics, pharmacokinetics

## Abstract

**Background:**

Trauma is the most common cause of death in children >1 year of age, with hemorrhage being the most common preventable cause of death after injury. Antifibrinolytics like tranexamic acid (TXA) are a key aspect of trauma management in children, but optimal dosing remains unknown. The objective of this study was to derive a TXA dose in children with trauma‐related bleeding that approximates the TXA exposure in adult trauma patients using a population pharmacokinetic (popPK) model from adults with severe traumatic injury.

**Study Design and Methods:**

Model‐based simulation was performed by extrapolating a previously published popPK model of TXA in adults with trauma‐related bleeding to pediatric patients. A virtual pediatric trauma population was simulated utilizing published covariate values and an allometrically scaled adult model applied to predict the TXA PK profile in children with trauma‐related bleeding.

**Results:**

An IV TXA bolus of 25 mg/kg (max 2 g) in children with trauma‐related bleeding approximates the C_max_ after a 2 g IV bolus in an adult trauma population. No dose from 20 to 35 mg/kg achieved the AUC_0–4h_ or AUC_0–8h_ that results from a 2 g IV bolus in adults.

**Discussion:**

In children with trauma‐related bleeding, a TXA 25 mg/kg IV bolus is predicted to approximate the C_max_ achieved with a 2 g IV bolus in adults. More frequent dosing may be necessary in children to achieve a similar total drug exposure as adults with trauma‐related bleeding.

**Abbreviations**
AUC: area under the curve
CPB: cardiopulmonary bypass
GFR: glomerular filtration rate
IL‐8: interleukin‐8
IV: intravenous
NCA: noncompartmental analysis
NIRS: near‐infrared spectroscopy
PK: pharmacokinetic
PLT: platelet count
popPK: population pharmacokinetic
TXA: tranexamic acid

## 1 INTRODUCTION

Trauma is the most common cause of death in children >1 year of age, with hemorrhage being the most common preventable cause of death after injury.^1^, ^2^ Mortality in children with life‐threatening bleeding from traumatic injury is 36%–50% compared to 20%–24% in adults.^3^, ^4^, ^5^, ^6^ Damage control resuscitation is a bundle of care aimed at improving outcomes in patients with life‐threatening trauma‐related bleeding.^7^, ^8^ Many aspects of damage control resuscitation have been studied in adults. Conversely, while many damage control resuscitation principles have been applied in pediatric trauma,^9^, ^10^, ^11^ clinical trials have not been performed.

Antifibrinolytics like tranexamic acid (TXA) are a key aspect of damage control resuscitation. TXA inhibits plasminogen and improves hemostasis via reduction in clot lysis. TXA may also impact the immune system and the endothelium, which contribute to improved survival.^12^ Multiple trials in adults with traumatic injury indicate improved survival with early use.^13^, ^14^ These trials have utilized multiple dosing regimens that are based on target TXA plasma concentrations derived from in vitro studies.

The use of TXA in children with life threatening bleeding from traumatic injury is variable with a large range of doses used clinically.^15^, ^16^, ^17^ The optimal dose of TXA in children with life‐threatening traumatic bleeding is not known and TXA lacks FDA labeling for this indication. There are also no published pharmacokinetic (PK) data for TXA in children with severe bleeding to guide dosing. TXA dosing in adults with severe trauma‐related bleeding has included both an intravenous (IV) bolus with subsequent 8 h maintenance infusion and IV bolus only dosing.^12^, ^18^, ^19^ There has been a trend to favor bolus only dosing based on trial data and logistical benefits to improve prehospital TXA use. For example, the US and UK militaries have changed guidelines to use a 2 g IV bolus dose.^20^, ^21^ The objective of this analysis is to perform TXA dose simulation for children based on population PK (popPK) modeling of data generated from adults with severe traumatic injury. We focused on IV bolus only dosing because adult data indicate achievement of the target plasma exposure without the need for a prolonged infusion, a parameter we expect would translate to children with severe trauma‐related bleeding.

## 2 MATERIALS AND METHODS

As this study included solely simulation utilizing published data, it did not fall under the institutional review board's guidelines as human subjects research. To perform pediatric TXA simulations, we scaled a previously published adult popPK model built with data collected during the Tranexamic Acid Mechanisms and Pharmacokinetics in Traumatic Injury (TAMPITI) trial.^12^ A virtual pediatric population was next created utilizing relevant covariate values for children from the literature. We then simulated TXA exposures in our virtual pediatric population using multiple different dosing regimens to target the exposure seen in adults with trauma‐related bleeding (see Supporting Information for additional detail).

### 2.1 Adult population PK model

A previously published adult popPK model of IV TXA was utilized for this simulation.^22^ Briefly, the adult popPK model was built utilizing a dataset with 597 plasma TXA samples from 94 TAMPITI participants (placebo participants excluded from analysis). The included participants received either a 2 g or 4 g IV TXA bolus. Platelet count (PLT), skeletal muscle oxygen saturation measured by near‐infrared spectroscopy (NIRS), and interleukin‐8 (IL‐8) concentration were included as covariates on TXA clearance. The final parameter estimates for this adult model are presented in Table 1.

**TABLE 1:** Parameter estimates for the adult popPK model.

Parameter	Point estimate	RSE	95% CI	Shrinkage
CL (mL/min/70 kg)^0.75^	192	10.2%	154 to 230	–
V1 (mL/70 kg)	17,300	4.94%	15,600 to 19,000	–
Q (mL/min/70 kg)^0.75^	80.1	7.12%	68.9 to 91.3	–
V2 (mL/70 kg)	11,400	4.31%	10,400 to 12,400	–
PLT on CL	0.468	14.6%	0.335 to 0.601	–
NIRS on CL	−0.29	15.7%	−0.379 to −0.201	–
IL‐8 on CL	−0.0887	25.9%	−0.134 to −0.0436	–
Inter‐individual variability				
CL	0.106	14.7%	0.0754 to 0.137	2.7%
V1	0.0688	31.3%	0.0267 to 0.111	13.4%
Q	0.879	29.5%	0.371 to 1.39	14.7%
V2	0.0589	36.5%	0.0168–0.101	15.1%
Residual error				
Proportional	0.0238	4.5%	0.0217 to 0.0259	21.5%

Abbreviations: CI, confidence interval; CL, clearance; IL‐8, interleukin 8; NIRS, near‐infrared spectroscopy; PLT, platelet count; Q, intercompartmental clearance; RSE, residual standard error; V1, central volume of distribution; V2, peripheral volume of distribution.

### 2.2 Adult TXA exposure simulation

The final adult popPK model and median TAMPITI participant covariate values at time 0 (Table 2) were utilized in simulations (*n* = 1000) to estimate the predicted TXA time‐concentration profile after a 2 g IV bolus dose. From these simulated adult PK profiles, we calculated the duration that the simulated plasma TXA concentration remained above 10 mg/L (*T* > 10 mg/L), which was used for comparison to simulated pediatric time‐concentration profiles in later steps. A concentration of 10 mg/L was chosen based on in vitro data suggesting that TXA concentrations of 10 mg/L provide 80% inhibition of fibrinolysis.^23^

**TABLE 2:** Covariate values utilized in TXA dose simulations.

Covariate	Adult^a^	Pediatric^b^
Age (years)	–	4.7–15.4
Weight (kg)	80.1	17.7–58.2
PLT (K/μL)	197	205–433
NIRS (%)	88	51–80
IL‐8 (pg/mL)	20.3	6.6–50.2

Abbreviations: IL‐8, interleukin 8; NIRS, near‐infrared spectroscopy; PLT, platelet count.

^a^ Median value at time 0 in TAMPITI Trial participants.

^b^ Range from which virtual population was constructed.

### 2.3 Pediatric virtual subjects

A population of 1000 virtual pediatric subjects was created utilizing covariate values (weight, PLT, NIRS, and IL‐8) from peer‐reviewed literature describing pediatric trauma patients, as shown in Table 2.^4^, ^24^, ^25^, ^26^, ^27^, ^28^ Briefly, the minimum and maximum allowable value for each covariate was set utilizing either the 25th and 75th percentile values (if reported as median [IQR]) or one standard deviation above and below the mean (if reported as mean [SD]) from the literature (Table 2). Random sampling from each of the covariate value ranges then was utilized to create 1000 unique virtual subjects.

### 2.4 Pediatric simulations

The adult popPK model was then scaled to children utilizing allometric scaling of weight, in which an exponent is applied to account for the impact of size on metabolic processes like drug clearance. An adult reference weight of 70 kg was used. Pediatric PK profiles were simulated utilizing TXA IV boluses of 20, 25, 30, and 35 mg/kg (max 2 g/dose) administered over 1 min. Predicted pediatric TXA exposure was estimated by calculating maximum concentration (C_max_), area under the curve (AUC) from 0 to 4 h, AUC from 0 to 8 h, and T > 10 mg/L for each virtual pediatric subject using a noncompartmental analysis (NCA). The results were then compared to the adult TXA exposures. Additional simulations were subsequently performed to explore the impact of administration rate on the predicted drug exposure, simulating administration times from 1 to 10 min. The objective was to approximate the adult AUC while maintaining a similar C_max_ due to a known dose‐toxicity relationship with TXA.^29^, ^30^

## 3 RESULTS

**FIGURE 1:**
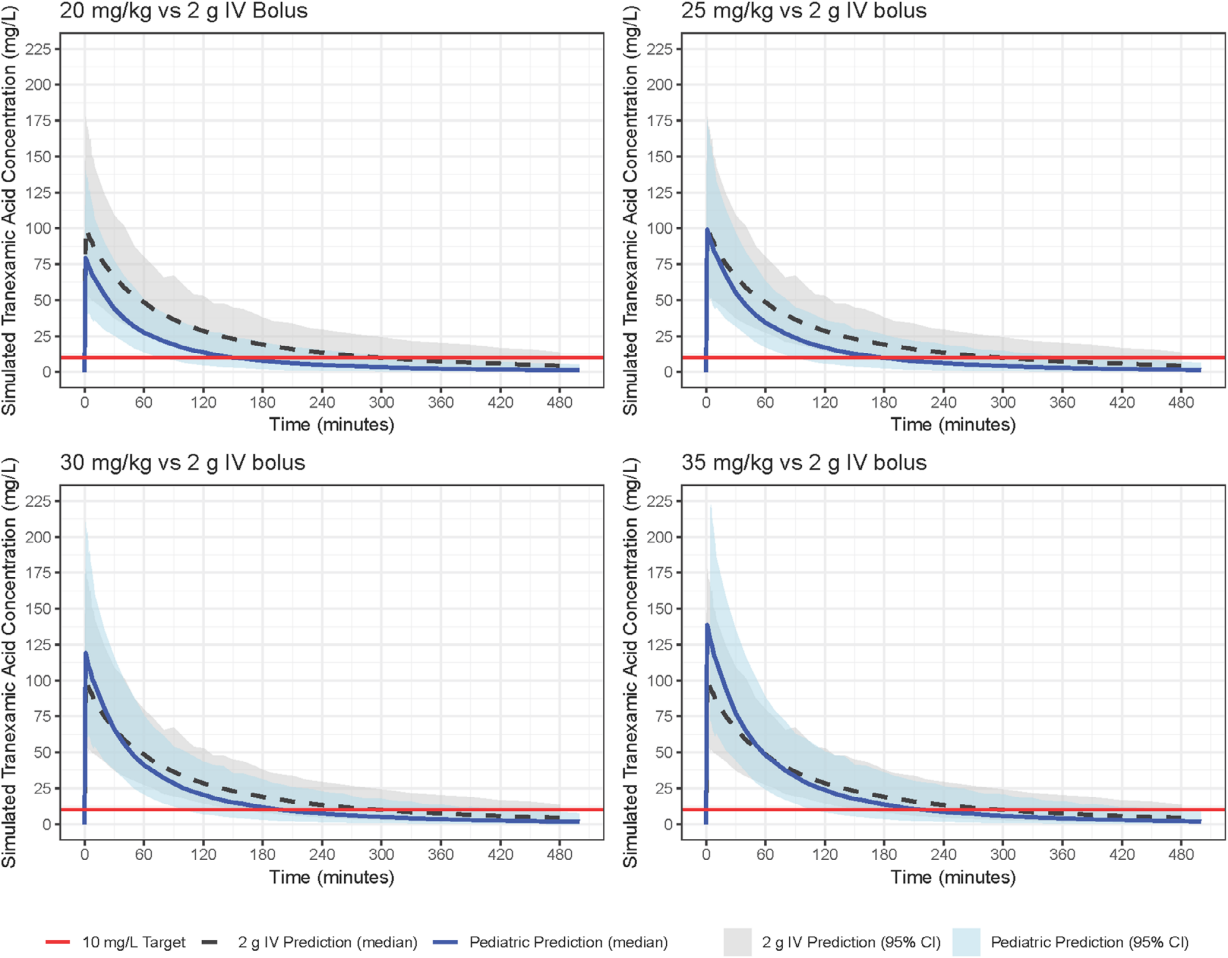
Time‐concentration plots for tranexamic acid bolus doses in children and adults.

The final model used for both adult and pediatric simulations is shown in Equations ((1), (2), (3), (4)):

(1) $$\text{TVCL}=\begin{array}{l} 190\times {\left(\mathrm{WT}/70\right)}^{0.75}\times {\left(\mathrm{PLT}/196\right)}^{0.468}\\ \times {\left(\text{NIRS}/88\right)}^{-0.29}\times {\left(\mathrm{IL}-8\right)}^{-0.0873}\times e^{0.106} \end{array}$$

(2) $$\text{TVV1}=17,300\times {\left(\mathrm{WT}/70\right)}^{1}\times e^{0.0688}$$

(3) $$\mathrm{TVQ}=80.1\times {\left(\mathrm{WT}/70\right)}^{0.75}\times e^{0.879}$$

(4) $$\text{TVV2}=11,400\times {\left(\mathrm{WT}/70\right)}^{1}\times e^{0.0589}$$

where TVCL is the typical value of CL, TVV1 is the typical value of V1, TVQ is the typical value of Q, and TVV2 is the typical value of V2. Time‐concentration plots for all simulated dosing regimens are shown in Figure 1.

### 3.1 Adult and pediatric simulated TXA exposures

Based on the simulated PK profile of a TXA 2 g IV bolus in adult trauma patients, plasma TXA concentration is expected to remain >10 mg/L for a median time of 5.27 h (Table 3). All simulated pediatric doses resulted in shorter times >10 mg/L compared to the adult simulation, with 35 mg/kg being the most similar time at 3.7 h.

**TABLE 3:** Pediatric versus adult model‐predicted TXA exposure.

Parameter	Adult 2 g	Pediatric 20 mg/kg	Pediatric 25 mg/kg	Pediatric 30 mg/kg	Pediatric 35 mg/kg
Time above 10 mg/L (h)	5.27	2.5 (2–3.3)	3 (2.3–4)	3.3 (2.5–4.3)	3.7 (2.8–4.8)
C_max_ (mg/L)	117.1 (98.9–139.7)	91.7 (77.4–109.6)	114.6 (96.7–137.0)	138.0 (116.0–164.0)	160.4 (135.4–191.8)
AUC_0–4h_ (mg*min/L)	8738 (7433‐10,347)	5012 (4099‐6196)	6265 (5124‐7744)	7515 (6147‐9294)	8770 (7173‐10,842)
AUC_0–8h_ (mg*min/L)	10,691 (8901‐12,959)	5676 (4473‐7211)	7096 (5591‐9013)	8513 (6710‐10,818)	9928 (7828‐12,618)

*Note*: All values reported as median (Q1–Q3) of each parameter.

Abbreviations: AUC, area under the curve; C_max_, maximum concentration.

### 3.2 Noncompartmental analysis results

Analysis of a TXA 2 g IV bolus in simulated adult trauma patients resulted in a C_max_ of 117.1 mg/L, AUC_0–4_ of 8738 mg*min/L, and AUC_0–8_ of 10,691 mg*min/L (Table 3). Pediatric simulations showed that a TXA bolus of 25 mg/kg is predicted to result in a C_max_ most similar to that in the adult simulation at 114.6 mg/L. Doses of 30 and 35 mg/kg resulted in C_max_ values exceeding those from a 2 g IV bolus dose in adults. No pediatric dose simulations resulted in an AUC value equal to or greater than that predicted with a 2 g IV bolus in adults. A dose of 35 mg/kg resulted in an AUC_0–4_ and AUC_0–8_ most similar to that predicted in the adult simulation. Extending the administration time from 1 min to 5 or 10 min minimally impacted predicted drug exposure (Table 4).

**TABLE 4:** Simulated TXA exposure: 25 mg/kg with varied administration time.

Parameter	1 min infusion	5 min infusion	10 min infusion
Time above 10 mg/L (h)	3 (2.3–4)	3 (2.3–4)	2.9 (2.2–4)
C_max_ (mg/L)	114.6 (96.7–137.0)	107.8 (90.8–130.1)	95.9 (79.8–114.8)
AUC_0–4h_ (mg*min/L)	6265 (5124–7744)	6259 (5117–7717)	6244 (5100–7699)
AUC_0–8h_ (mg*min/L)	7096 (5591–9013)	7088 (5594–9013)	7087 (5589–9009)

*Note*: All values reported as median (Q1–Q3) of each parameter.

Abbreviations: AUC, area under the curve; C_max_, maximum concentration.

## 4 DISCUSSION

We derived TXA dosing in pediatric trauma through extrapolation and dose simulation utilizing a model of TXA in adult trauma patients. The goal was to determine a pediatric dose that approximates the adult AUC while maintaining a similar C_max_ value to avoid excessive toxicity. None of the median AUC_0–4_ or AUC_0–8_ values for the pediatric doses simulated matched the adult estimates. A 35 mg/kg bolus dose came closest to matching the exposure estimated in adults after a 2 g bolus dose; however, this resulted in excessively high C_max_ values. The C_max_ achieved with a 25 mg/kg TXA bolus in our virtual pediatric population was similar to that predicted in adults receiving a 2 g IV bolus.

TXA PK is predicted to be different in children compared to adults for two primary reasons. The first is due to the allometric scaling of clearance. Utilizing an established allometric exponent of 0.75 on clearance, as weight decreases, TXA clearance is likewise expected to decrease relative to the adult reference weight. For every 10 kg decrease in simulated subject weight, TXA clearance is predicted to decrease by 10%–15%. However, as PLT and IL‐8 increase, TXA clearance is likewise predicted to increase. For every 25 K/μL increase in PLT count, TXA clearance is predicted to increase by 5%–10%. Considering these relationships, the predicted TXA clearance in these simulations is highly dependent on the covariate values utilized. In our simulations, it is likely that the higher PLT values utilized relative to the TAMPITI participants outweighed the effect of allometric scaling, resulting in overall faster TXA pediatric clearance compared to the adult simulation.

The PLT, NIRS, and IL‐8 values utilized in the adult TXA simulations came directly from TAMPITI participants, the population on which the adult model is based. For corresponding pediatric covariate values, there are few data in the literature to inform selection. The values selected here come from a mixture of populations including children who sustained blunt trauma and those presenting to combat support hospitals. These children had a lower rate of penetrating trauma compared to TAMPITI trial participants. Additionally, the adult NIRS value represents the median admission NIRS in TAMPITI participants, while only the median lowest NIRS value recorded was available in children. Based on the relationship seen in the TAMPITI model, these lower pediatric NIRS values result in a higher rate of TXA clearance. While these covariate values resulted in median pediatric AUC values lower than those predicted in the adult simulation, it is also notable that the interquartile ranges of AUC values of the 25, 30, and 35 mg/kg doses overlap with that of the adult simulation.

Altering administration time from 1 min to 5 or 10 min minimally impacted predicted drug exposure. This is likely due to the rapid pediatric clearance predicted, minimizing the effect of prolonged administration. Administration times >10 min were not explored as clinical practicality in a trauma activation was considered vital. To maximize clinical utility and ease of administration, shorter administration time with the ability to re‐dose if necessary was prioritized over maximized single dose exposure.

The adult TXA model used for this extrapolation is similar to other published models in adult trauma patients^31^, ^32^ and healthy volunteers.^33^ There are currently no published PK models of TXA in pediatric trauma patients. Several popPK models have been published in children undergoing craniosynostosis or cardiac surgeries, but extrapolation from these to the trauma population is not straightforward. Gertler et al. modeled TXA in neonates and infants undergoing cardiac surgery. Dosing included a 50 mg/kg IV bolus plus 50 mg/kg added to the cardiopulmonary bypass (CPB) prime solution. A 2‐compartment model best described the data, with significant covariates of body weight, CPB, and post‐menstrual age. The V1 parameter estimate in this model is similar to the present study, with other parameter estimates differing (CL = 63 mL/min/70 kg, V1 = 13,600 mL/70 kg, Q = 272 mL/min/70 kg, V2 = 18,000 mL/70 kg).^34^ Goobie et al. modeled TXA in pediatric patients undergoing craniosynostosis surgery.^35^ Participants received TXA 50 mg/kg IV over 15 min plus 5 mg/kg/h during surgery. A 2‐compartment model best described TXA disposition, with weight and age as covariates on CL. Weight was also a covariate on V1, with no covariates on Q or V2. CL and V1 point estimates are similar to our model, while weight was not a covariate on Q or V2 (CL = 223.5 mL/min/70 kg, V1 = 13,650 mL/70 kg, Q = 46.2 mL/min, V2 = 1530 mL). Wesley et al. performed popPK modeling in neonates, infants, and children undergoing cardiac surgery with CPB.^36^ Participants received TXA 100 mg/kg IV over 5 min plus 10 mg/kg/h during surgery and 100 mg/kg added to the CPB prime. Parameter estimates were significantly different than the present model (CL = 910 mL/min/70 kg, V1 = 3080 mL/70 kg, Q = 7840 mL/min/70 kg, V2 = 88,200 mL/70 kg). Being derived from populations with different pathophysiology than severe trauma‐related bleeding, it is difficult to compare these results to our model. To put these differences in perspective, Grassin‐Delyle et al. performed popPK modeling of TXA in adult patients undergoing CPB. TXA was again best described by a 2‐compartment model, with parameter estimates also dissimilar to the present study (CL = 80 mL/min/70 kg, V1 = 6600 mL/70 kg, Q = 536.7 mL/min/70 kg, V2 = 10,800 mL/70 kg).^37^

The studies from which these pediatric models were derived utilized higher loading doses than our simulations, ranging from 50 mg/kg to 100 mg/kg. These higher loading doses resulted in higher C_max_ values than those predicted in our simulations, which were not necessarily associated with improved hemostasis, as demonstrated by Goobie et al. in their double‐blind randomized trial of high versus low‐dose TXA in pediatric craniosynostosis surgery.^38^ A high‐dose regimen utilizing a 50 mg/kg IV TXA bolus plus 5 mg/kg/h was compared to a low‐dose regimen utilizing a 10 mg/kg IV TXA bolus plus 5 mg/kg/h. The high‐dose group had a mean (SEM) C_max_ of 199.4 mg/L (10.6) compared to 67.5 mg/L^14^ in the low‐dose group, with both groups maintaining TXA concentrations >10 mg/L throughout surgery. There were no significant differences in clinical outcomes between groups including intraoperative blood loss, total red blood cell transfusion, intraoperative or post‐operative day 1 laboratory markers, or ICU or hospital length of stay. No adverse outcomes were reported. In the study by Gertler et al. discussed previously, the mean (SD) C_max_ achieved was 213 mg/L (54) and one infant experienced 3 seizures on the day of TXA administration.^34^ In light of these data and TXA's demonstrated dose‐toxicity relationship,^29^, ^30^ it may be pertinent to favor a dosing strategy emphasizing a similar C_max_ in children and adults, with consideration of additional doses in children with trauma‐related bleeding due to the predicted faster clearance.

There are several limitations with the present study, including the use of an adult popPK model to extrapolate dosing for children. The simulated pediatric subjects fall outside the dataset used to construct the model; therefore, the assumption that PK relationships can be predicted based on established allometric relationships requires clinical validation. This study also assumes the covariates found to significantly impact adult TXA PK are the same for children, which may not be the case. The lack of robust pediatric covariate information for dose simulations introduces additional assumptions that likewise require validation. The pediatric population simulated in this study included children as young as 4.6 years. At this age, glomerular filtration rate (GFR) is expected to meet or exceed adult values.^39^ However, as primarily a renally eliminated drug, it is important to account for maturational changes in GFR and subsequent TXA clearance for children less than 1 year of age.^40^

## 5 CONCLUSION

The optimal dosing regimen for TXA in pediatric trauma patients remains unclear. In this study, there were differences in predicted total plasma exposure and maximum concentration between children and adults across multiple TXA dosing regimens. While a 25 mg/kg bolus in a pediatric trauma patient is predicted to achieve a similar C_max_ as a 2 g IV bolus in adult trauma patients, no pediatric dosing regimen less than 35 mg/kg was predicted to achieve an AUC_0–4_ or AUC_0–8_ equal to a TXA 2 g bolus in adult trauma patients. Therefore, unique pediatric dosing regimens may be warranted. Further study is needed to assess the effectiveness and safety of TXA dosing in children.

## FUNDING INFORMATION

Funding was provided by the Center for the Biomedical Advanced Research and Development Authority (Contract 75A50123C00047). Kevin Downes is supported by the Eunice Kennedy Shriver National Institute of Child Health & Human Development of the National Institutes of Health under Award Number K23‐HD091365.

## CONFLICT OF INTEREST STATEMENT

The authors have disclosed no conflicts of interest.

## Supporting information


**Data S1.** Supporting Information.

## Data Availability

The data that support the findings of this study are available from the corresponding author upon reasonable request.
