# Supplementary material for: Tranexamic acid dosing in pediatric trauma: Dose simulation based on population pharmacokinetic modeling in adult trauma patients
Source: Transfusion. 2025 Dec 23;66(Suppl 1):S257–65. doi: 10.1111/trf.70047 (PMC12794529; doi:10.1111/trf.70047)
Supplement: Supplementary file 1 — Data S1. Supporting Information. [file TRF-66-S257-s001.docx]

**Materials and Methods Supplementary Information**

*Adult Population PK Model*

All parameters were allometrically scaled using the model $TVP= \theta_{TVP}\times{(\frac{{WT}_{i}}{{WT}_{ref}})}^{\theta_{allometric}}$, where TVP is the scaled typical value of the parameter (i.e., clearance or volume of distribution), θ_TVP_ is the original typical value of the parameter of interest, WT_i_ is the weight of individual *i*, and WT_ref_ is a reference weight (i.e., 70 kg). This is similar to linearly scaling parameters based on weight, but additionally utilizes an allometric exponent that accounts for the impact of size on metabolic processes.(23) $\theta$_allometric_ was fixed at 0.75 for clearance parameters and 1 for volume parameters.

*Adult TXA Exposure Simulation*

Noncompartmental analysis (NCA) was performed to quantify these simulated adult TXA exposures. Maximum concentration (C_max_), area under the curve (AUC) from 0-4 hours, and AUC from 0-8 hours were calculated for each simulated subject with the linear up log down method in PKanalix 2023R1 (Lixoft SAS, a Simulations Plus company). Two different AUCs were calculated to compare TXA exposure. The time from 0-4 hours is the period when bleeding is likely to be most severe and approximates the duration that the plasma concentration of TXA is expected to remain above 10mg/L after a single bolus dose. The period from 0-8 hours matches the total time included in the published bolus/infusion regimen listed in tertiary dosing references.(25)

*Pediatric Virtual Subjects*

To derive the distribution of pediatric weights to be used, virtual subject ages were first derived from the trauma cohort of the Massive Transfusion in Children (MATIC-1) study.(15) MATIC-1 was a prospective observational study of children who received >40mL/kg of total blood products over 6 hours or had a massive transfusion protocol activation. These MATIC-1 ages were translated to virtual subject weights by finding the 50^th^ percentile weight-for-age according to CDC growth tables for the 25^th^ and 75^th^ percentile ages in MATIC-1. The numeric bounds for all other covariates were set utilizing either the 25^th^ and 75^th^ percentile values (if reported as median [IQR]) or one standard deviation above and below the mean (if reported as mean [SD]) (**Table 2**). Random sampling from each of the covariate value ranges then was utilized to create 1,000 unique virtual subjects.

*Scaling Adult Population PK Model to Children*

The adult popPK model was allometrically scaled to children utilizing weight and the previously described allometric equation. The value of θ_allometric_ was fixed at 0.75 for clearance terms and 1 for volume terms. The same reference weight of 70kg that was utilized in the adult model was used here for scaling.
